# Screening for intimate partner violence during pregnancy: a test accuracy study

**DOI:** 10.1093/eurpub/ckac009

**Published:** 2022-02-03

**Authors:** Antonella Ludmila Zapata-Calvente, Jesús L Megías, Casilda Velasco, Africa Caño, Khalid S Khan, Leticia Rubio, Stella Martín-de-las-Heras

**Affiliations:** Brain and Behavior Research Center (CIMCYC), University of Granada, Granada, Spain; Department of Experimental Psychology, University of Granada, Granada, Spain; Department of Nursing and Midwifery, University of Jaen, Jaen, Spain; Department of Obstetrics and Gynecology, University Hospital, Granada, Spain; Department of Preventive Medicine and Public Health, University of Granada, Granada, Spain; CIBER of Epidemiology and Public Health (CIBERESP), Granada, Spain; Department of Forensic Medicine, University of Malaga, Malaga, Spain; Department of Forensic Medicine, University of Malaga, Malaga, Spain

## Abstract

**Background:**

Intimate partner violence (IPV) against women is a serious health problem that affects pregnancy more frequently than other obstetric complications usually evaluated in antenatal visits. We aimed to estimate the accuracy of the Women Abuse Screening Tool-Short (WAST-Short) and the Abuse Assessment Screen (AAS) for the detection of IPV during and before pregnancy.

**Methods:**

Consecutive eligible mothers in 21 public primary health antenatal care centres in Andalusia (Spain) who received antenatal care and gave birth during January 2017–March 2019, had IPV data gathered by trained midwives in the first and third pregnancy trimesters. The index tests were WAST-Short (score range 0–2; cut-off 2) and AAS (score range 0–1; cut-off 1). The reference standard was World Health Organization (WHO) IPV questionnaire. Area under receiver operating characteristics curve (AUC), sensitivity and specificity with 95% confidence intervals (CI) were estimated for test performance to capture IPV during and before pregnancy, and compared using paired samples analysis.

**Results:**

According to the reference standard, 9.5% (47/495) and 19.4% (111/571) women suffered IPV during and before pregnancy, respectively. For capturing IPV during pregnancy in the third trimester, the WAST-Short (AUC 0.73, 95% CI 0.63, 0.81), performed better than AAS (AUC 0.57, 95% CI 0.47, 0.66, *P* = 0.0001). For capturing IPV before pregnancy in the first trimester, there was no significant difference between the WAST-Short (AUC 0.69, 95% CI 0.62, 0.74) and the AAS (AUC 0.69, 95% CI 0.62, 0.74, *P* = 0.99).

**Conclusions:**

The WAST-Short could be useful to screen IPV during pregnancy in antenatal visits.

## Introduction

Intimate partner violence (IPV) against women, defined as any act of physical, sexual, psychological violence or controlling behaviours committed by a partner or ex-partner,^1^ is a serious health problem that affects pregnancy more frequently than other obstetric complications usually evaluated in prenatal visits.^2^^,^^3^ It is a risk factor for ill-health of the mother and the offspring,^4^^,^^5^ and its prevalence in pregnancy is 28.4%, 13.8% and 8.0% for emotional, physical and sexual abuse respectively,^6^ with variation between countries.^1^^,^^7^

Many screening instruments exist,^8–10^ but few have been validated in pregnant women. Most of the studies using screening tools in pregnant women have not collected prospective data and they have administrated the screening tool at a single time point during the pregnancy^11–14^ or in the postpartum period.^7^^,^^15–17^ Some of them have been conducted in specific cultural contexts,^12–15^^,^^18^^,^^19^ limiting generalizability. Other limitations include deficiencies such as not reporting the sensitivity or specificity values of the screening tools,^7^^,^^11–14^^,^^16^ finding very low sensitivity values^17^^,^^20^ or not using validated IPV tools as reference standards.^19^

Therefore, we estimated the accuracy of screening by two of the most commonly used instruments: the Woman Abuse Screening Tool-Short (WAST-Short)^8^ and the Abuse Assessment Screen (AAS)^21^ for the detection of IPV during and before pregnancy. We selected the WAST-Short not only because with two items is the shortest screening test to detect IPV in the healthcare context, but also because it has shown good sensitivity properties;^9^^,^^22^ however, there is less evidence of its performance in pregnant women. The AAS was chosen because it was developed to be applied in antenatal care, however the evidence of its accuracy is mixed.^20^^,^^23^

As reference standard we used the World Health Organization (WHO) IPV questionnaire (for emotional, physical and sexual abuse) administered in the third and first trimesters, respectively. We chose this instrument as a reference standard as it complies with the WHO’s methodological and ethical guidelines for research on violence against women,^24^ it helps to solve the problem of non-comparability between studies^25^ and it has good psychometric properties.^26^^,27^ Besides, this instrument has been used previously in several studies with samples of pregnant women.^28^

## Methods

The study protocol was approved by the research ethics committees of all participating centres (Research Ethics Committees of Healthcare Centres, Healthcare Counselling, Andalusian Healthcare Service, Andalusian Government, Spain. Protocol code: VIO-EMB-AP-2017. Internal code: E.C 41/2017. Signed on 27 September 2017). All participants provided verbal and written informed consent prior to enrolment. There were neither formal patient involvement nor core outcome sets in the design of this research as the protocol predated the introduction of these initiatives.

### Study participants

A total of 730 consecutive eligible women in 21 public primary healthcare antenatal centres in Andalusia (Spain) were invited to participate in the study in their first antenatal visit in during January 2017–March 2019. The final sample consisted of 592 women who agreed to participate in the study, representing 81.04% of the sample.

### Data collection procedure and instruments

Midwives involved in the care of the participants were specifically trained to collect the study data in the first and the third trimester visits in one-to-one interviews. Strict anonymity and confidentiality were guaranteed. Women participating provided signed informed consent on enrolment. Information concerning the police, judicial and social services and resources was provided to all participants. Clinicians providing care to participants were unaware of the findings of the tests used in the study.

#### Reference standard

We used version 11 of the questionnaire from the *WHO Multi-Country Study on Women’s Health and Domestic Violence Against Women*^29^ that has been adapted for the Spanish population.^30^ It uses 15 items to measure emotional (5 items; e.g. ‘Did he insult you or made you feel bad about yourself?’), physical (6 items; e.g. ‘Did he pushed, grabbed or pulled your hair?’) and sexual (4 items, e.g. ‘Did he forced you to have sex when you didn’t want to?’) violence within a common classification that enables standardization and cross-country comparisons. Women answered these questions using on a 5-point scale (1 = never, 2 = once, 3 = sometimes, 4 = many times, 5= No answer). We created five prevalence indicators: WHO emotional, WHO physical, WHO sexual, WHO global IPV (women who have suffered at least one of the behaviours that are described within each of the three types of violence) and WHO physical–sexual IPV (considering only women who have suffered at least one act of physical or sexual IPV).

#### Screening instruments (Index tests; measured in the first and third trimesters)

We used two instruments to screen for IPV before and during the pregnancy period:

##### WAST-Short

The WAST-Short is an IPV screening tool which contains the first two items of the eight-item WAST to assess IPV and has been effective in identifying abuse in women visiting family physicians for periodic health examinations, prenatal care or treatment for illness.^8^ The WAST-Short assesses the degree of relationship tension and difficulty in resolving arguments within a relationship. It contains two questions: ‘In general, how would you describe your relationship?’ and ‘Do you and your partner work out arguments with…?’ Responses ranged from 1 (a lot of tension or great difficulty) to 3 (no tension or no difficulty). Their sensibility and specificity were analyzed in the Spanish healthcare context several years ago,^22^ but not specifically with pregnant women. We used one of the scoring rules tested in the English study;^8^ the one that gave better results in the Spanish validation of the original version^31^ and in posterior studies developed in Spain.^22^ In the method, a score of 1 was assigned to all positive responses (e.g. some or a lot of tension) with negative responses assigned a score of 0, giving overall scores ranging from 0 to 2. Scores of 2 were considered to be positive for the purposes of screening.

##### AAS^21^

The AAS was developed for application in antenatal care, and it is available in both English and Spanish. The AAS contains four questions on emotional, physical and sexual abuse at any time during the woman’s life, within the previous year and during pregnancy. It also asks about the relationship with the aggressor, the frequency of the violence, any fear of the perpetrator and the severity of physical violence. For the purpose of this study, we added the instruction ‘before pregnancy’ (in the first prenatal visit) and ‘since you are pregnant’ (in the third prenatal visit) and we focused only the three questions that have a correspondence with the reference standard: emotional (e.g. ‘Have you been humiliated, insulted, belittled, threatened or caused any other emotional harm?’), physical (e.g. ‘Have you been pushed, hit, slapped, kicked or physically injured?’) and sexual (e.g. ‘Have you been forced to have sex?’) abuse where we could identify the perpetrator. A score of 1 was assigned to the item if the women said ‘yes’ and 0 if she said ‘no’. A positive response to any one of these questions indicates that the responder may be a victim of abuse. We created five scores: AAS emotional, AAS physical, AAS sexual, AAS global (if women have suffered at least one act of emotional, physical or sexual IPV) and AAS physical–sexual (if women have suffered at least one act of physical or sexual IPV). It has been recently validated in Spain,^20^ and data from five large and ethnically heterogeneous studies support its reliability and validity.^21^

## Statistical analyses

Descriptive statistics (including means, standard deviations, frequencies and percentages) were calculated for the socio-demographic characteristics of the sample. The complete dataset, excluding the missing values, was analyzed. The correlations between the WAST-Short, the AAS and the WHO IPV (global and subscales) are present in Supplementary table S1.

The sensitivity and specificity for the WAST-Short and the AAS were calculated at several cut-off scores against the WHO IPV subscales (as reference standards) in the first and third trimester to determine which score performed better in the classification of victims and non-victims of IPV in our sample of Spanish pregnant women, given that the evidence is mixed (see Discussion). A receiver operating characteristic (ROC) analysis was carried out to assess the overall accuracy of the WAST-Short and the AAS, using IPV-WHO as the criterion; this method allows display of all the pairs of sensitivity and specificity values achievable as the threshold is changed from low to high scores plotting the true-positive rate (sensitivity) on the vertical axis and the false-positive rate (one minus specificity) on the horizontal axis. The AUC is a quantitative indicator of the information content of a test, and it may be interpreted as an estimate of the probability that an abused woman chosen at random will, at each threshold, have a higher test score than a non-abused mother. The area under the ROC curve ranges from 0 to 1 with higher values indicating better accuracy.

## Results

In our sample (Supplementary figures S1 and S2 and table S2), 19.4% of pregnant women (*n *=* *111) reported having suffered at least one act of emotional, physical or sexual violence before pregnancy (3.5% missing values) and 9.5% (*n *=* *47) during pregnancy from a current partner according to the WHO questionnaire (16.4% missing values). We found a higher presence of emotional, followed by physical IPV and sexual violence before (19.3%, 5.4% and 2.4%, respectively) and during pregnancy (9.3%, 1.2% and 1%, respectively, Supplementary table S3). The mean age of women was 31.82 (SD = 5.61).

### Accuracy of screenings tools

#### IPV during pregnancy

**Figure 1:**
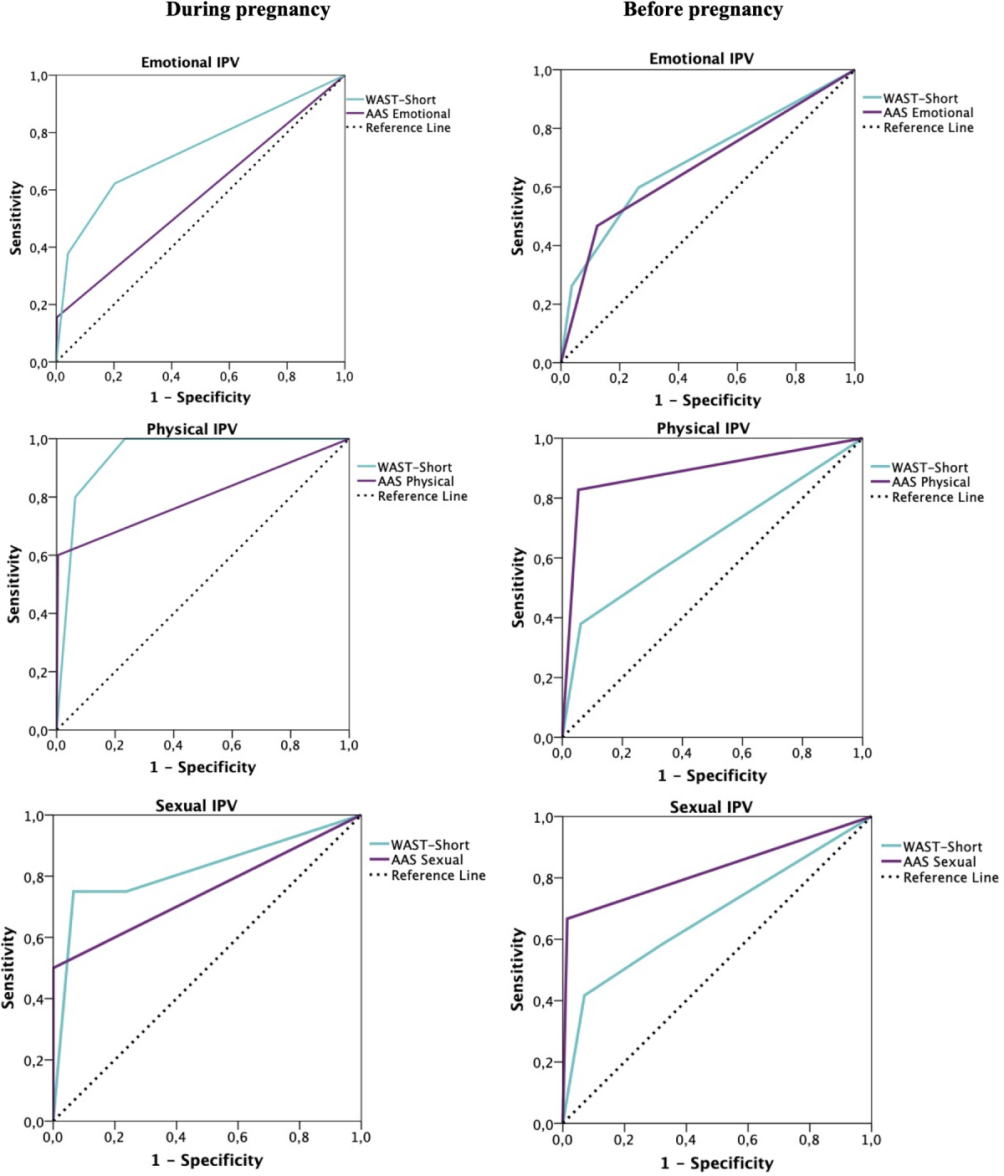
A receiver operating characteristic (ROC) curve of the short version of the woman abuse screening tool-short and the abuse assessment screen (AAS) using the World Health Organization questionnaires for emotional, physical and sexual intimate partner violence as reference standards during pregnancy (right column) and before pregnancy (left column)

The overall accuracy of the WAST-Short and the AAS, as screening instruments, can be described as the area under its ROC curve. The area under ROC curve (AUC) for global IPV (emotional, physical or sexual) was 0.73 for the WAST-Short and 0.57 for the AAS (table 1). For the combination of physical–sexual IPV, the AUC increased to 0.85 and 0.75 for the WAST-Short and for the AAS, respectively. Separating the results by type of IPV (figure 1, table 1), the AUC for emotional, physical and sexual IPV was found to be 0.74, 0.95 and 0.82 for the WAST-Short A and 0.58, 0.80 and 0.75 following AAS. The results indicated that in all the cases the lowest sum of false-positives and false-negatives was 0.5 in the WAST-short and the AAS. Given that the WAST-Short and the AAS scores were integral, 1 could be the most appropriate cut-off point to identify women suffering IPV with these screening tools. Using these cut-off points, the WAST-Short accurately categorized 62.2%, 100% and 75% of the women victims of emotional, physical and sexual IPV and between 60.9% and 83.3% of the women victims of global IPV and physical–sexual IPV, respectively. These numbers dropped to 16% (emotional), 67% (physical) and 60% (sexual), 17% (global IPV) and 50% (physical–sexual IPV) of victims with the AAS. Finally, the percentage of non-victims of emotional, physical, sexual, global IPV and physical–sexual IPV identified by the WAST-Short were 79.7%, 76.7%, 76.3%, 79.7% and 76.7%, respectively (table 1), while according to the AAS the identification of non-victims varies between 99.1% and 99.8% for all types of IPV. The comparison of the ROC curves showed that WAST-Short performed better than AAS (*P *=* *0.0001) to detect global IPV exposure (physical, sexual or emotional) in the third trimester (table 3). The comparison of the ROC curves for each type of IPV during pregnancy is shown in table 3.

**Table 1:** Area under the curve (AUC), sensitivity and specificity of WAST-Short and AAS for emotional, physical and sexual intimate partner violence (IPV) during pregnancy

Screening tools	Threshold scores	Type of IPV (reference standard: WHO definition)									
		IPV during pregnancy (captured in third trimester)									
		Emotional		Physical		Sexual		Global IPV		Physical/sexual IPV	
		Sen (CI)	Sp (CI)	Sen (CI)	Sp (CI)	Sen (CI)	Sp (CI)	Sen (CI)	Sp (CI)	Sen (CI)	Sp (CI)
WAST-Short	0.5	0.62 (0.47, 0.76)	0.80 (0.76, 0.83)	1 (0.46, 1)	0.77 (0.73, 0.80)	0.75 (0.22, 0.99)	0.76 (0.72, 0.80)	0.61 (0.45, 0.74)	0.80 (0.76, 0.83)	0.83 (0.36, 0.99)	0.77 (0.73, 0.80)
	1.5	0.38 (0.24, 0.53)	0.96 (0.93, 0.97)	0.80 (0.30, 0.99)	0.94 (0.91, 0.95)	0.75 (0.22, 0.99)	0.93 (0.91, 0.95)	0.37 (0.24, 0.52)	0.96 (0.93, 0.97)	0.67 (0.24, 0.94)	0.94 (0.91, 0.95)
	AUC (CI)	0.74^1^ (0.65, 0.83)		0.95^2^ (0.89, 0.99)		0.82^3^ (0.55, 1)		0.73^4^ (0.64, 0.82)		0.85^5^ (0.66, 1)	
AAS^a^	0.5	0.17 (0.08, 0.32)	0.99 (0.99, 1)	0.67 (0.24, 0.94)	0.99 (0.98, 1)	0.60 (0.17, 0.93)	1 (0.99, 1)	0.17 (0.07, 0.30)	0.99 (0.98, 1)	0.50 (0.20, 0.88)	0.99 (0.98, 1)
	AUC (CI)	0.58^1^ (0.50, 0.67)		0.80^2^ (0.53, 1)		0.75^3^ (0.43, 1)		0.57^4^ (0.48, 0.67)		0.75^5^ (0.49, 1)	

Notes: Sample *N *=* *592. WHO, World Health Organization; WAST-Short, Woman Abuse Screening Tool-Short; AAS, Abuse Assessment Screen; Sen, sensitivity; Sp, specificity; CI, confident intervals. Global IPV: having suffered at least one act of emotional, physical or sexual intimate partner violence; Global IPV (Phy + Sex): having suffered at least one act of physical or sexual intimate partner violence; WAST-Short: a score of 1 was assigned to all positive responses (e.g. some or a lot of tension) with negative responses assigned a score of 0, giving overall scores ranging from 0 to 2. Scores of 2 were considered to be positive for the purposes of screening. AAS: a positive response to any one of these questions indicates that the responder may be a victim of abuse. WAST-Short A: ^1^SD = 0.046, Asymp.sig.* *=* *0.000; ^2^SD = 0.025, Asymp.sig.* *=* *0.001; ^3^SD = 0.139, Asymp.sig.* *=* *0.027; ^4^SD = 0.046, Asymp.sig.* *=* *0.000; ^5^SD = 0.096, Asymp.sig.* *=* *0.003. AAS: ^1^SD = 0.049, Asymp.sig.* *=* *0.090; ^2^SD = 0.137, Asymp.sig.* *=* *0.022; ^3^SD = 0.162, Asymp.sig.* *=* *0.085; ^4^SD = 0.049, Asymp.sig.* *=* *0.099; ^5^SD = 0.132, Asymp.sig.* *=* *0.037.

a AAS emotional is compared with emotional IPV WHO; AAS physical is compared with physical IPV WHO; AAS sexual is compared with sexual IPV WHO; AAS global (emotional, physical or sexual) is compared with Global IPV WHO; AAS physical and sexual combined is compared with Global IPV WHO (physical and sexual).

**Table 2:** Area under the curve (AUC), sensitivity and specificity of WAST-Short and AAS for emotional, physical and sexual intimate partner violence (IPV) before pregnancy

Screening tools	Threshold scores	Type of IPV (reference standard: WHO definition)										
		**IPV previous pregnancy (captured in first trimester)**										
		Emotional		Physical		Sexual		Global IPV		Physical/sexual IPV		
		Sen (CI)	Sp (CI)	Sen (CI)	Sp (CI)	Sen (CI)	Sp (CI)	Sen (CI)	Sp (CI)	Sen (CI)	Sp (CI)	
WAST-Short	0.5	0.60 (0.50, 0.69)	0.74 (0.69, 0.77)	0.55 (0.36, 0.73)	0.69 (0.64, 0.72)	0.58 (0.29, 0.83)	0.68 (0.64, 0.72)	0.59 (0.49, 0.68)	0.73 (0.69, 0.77)	0.58 (0.39, 0.75)	0.69 (0.65, 0.73)	
	1.5	0.26 (0.18, 0.36)	0.96 (0.94, 0.98)	0.38 (0.21, 0.58)	0.94 (0.91, 0.96)	0.42 (0.16, 0.71)	0.93 (0.90, 0.95)	0.26 (0.18, 0.35)	0.96 (0.94, 0.98)	0.38 (0.22, 0.58)	0.94 (0.92, 0.96)	
	AUC (CI)	0.69^1^ (0.62, 0.75)		0.66^2^ (0.54, 0.78)		0.68^3^ (0.50, 0.86)		0.69^4^ (0.63, 0.75)		0.65^5^ (0.56, 0.75)		
AAS^a^	0.5	0.47 (0.38, 0.57)	0.88 (0.84, 0.90)	0.84 (0.65, 0.94)	0.95 (0.92, 0.96)	0.64 (0.35, 0.86)	0.99 (0.97, 0.99)	0.51 (0.42, 0.60)	0.86 (0.83, 0.89)	0.80 (0.67, 0.94)		0.94 (0.92, 0.96)
	AUC (CI)	0.67^1^ (0.61, 0.73)		0.88^2^ (0.81, 0.97)		0.82^3^ (0.66, 0.99)		0.69^4^ (0.63, 0.75)		0.87^5^ (0.80, 0.95)		

Notes: Sample *N *=* *592. WHO, World Health Organization; WAST-Short, Woman Abuse Screening Tool-Short; AAS, Abuse Assessment Screen; Sen, sensitivity; Sp, specificity; CI, confident intervals. Global IPV: having suffered at least one act of emotional, physical or sexual intimate partner violence. Global IPV (Phy + Sex): having suffered at least one act of physical or sexual intimate partner violence. WAST-Short: a score of 1 was assigned to all positive responses (e.g. some or a lot of tension) with negative responses assigned a score of 0, giving overall scores ranging from 0 to 2. Scores of 2 were considered to be positive for the purposes of screening. AAS: a positive response to any one of these questions indicates that the responder may be a victim of abuse. WAST-Short A: ^1^SD = 0.031, Asymp.sig.* *=* *0.000; ^2^SD = 0.061, Asymp.sig.* *=* *0.003; ^3^SD = 0.093, Asymp.sig.* *=* *0.035; ^4^SD = 0.031, Asymp.sig.* *=* *0.000; ^5^SD = 0.050, Asymp.sig.* *=* *0.001. AAS: ^1^SD = 0.032, Asymp.sig.* *=* *0.000; ^2^SD = 0.041, Asymp.sig.* *=* *0.000; ^3^SD = 0.083, Asymp.sig.* *=* *0.000; ^4^SD = 0.031, Asymp.sig.* *=* *0.000; ^5^SD = 0.038, Asymp.sig.* *=* *0.000.

a AAS emotional is compared with emotional IPV WHO; AAS physical is compared with physical IPV WHO; AAS sexual is compared with sexual IPV WHO; AAS global (emotional, physical or sexual) is compared with Global IPV WHO; AAS physical and sexual combined is compared with Global IPV WHO (physical and sexual).

**Table 3:** Comparison of ROC curves of WAST-Short and AAS for emotional, physical, sexual and global intimate partner violence (IPV) before and during pregnancy

	IPV previous pregnancy (captured in first trimester)			IPV during pregnancy (captured in third trimester)		
Type of IPV (reference standard: WHO definition)	AUC		Difference between AUC (CI)	AUC		Difference between AUC (CI)
	WAST-Short	AAS^a^		WAST-Short	AAS^a^	
Emotional	0.69	0.67	0.018, SE* *=* *0.036 (−0.05, 0.09), *P *=* *0.60	0.73	0.58	0.159, SE* *=* *0.039 (0.08, 0.24), *P *=* *0.0001
Physical	0.66	0.89	0.226, SE* *=* *0.071 (0.08, 0.36), *P *=* *0.0017	0.94	0.80	0.147, SE* *=* *0.110 (−0.06, 0.36), *P *=* *0.18
Sexual	0.68	0.83	0.149, SE* *=* *0.104 (−0.05, 0.35), *P *=* *0.15	0.82	0.75	0.070, SE* *=* *0.134 (−0.19, 0.33), *P *=* *0.59
Global IPV	0.69	0.69	0.0002, SE* *=* *0.036 (−0.07, 0.07), *P *=* *0.99	0.73	0.57	0.154, SE* *=* *0.039 (0.07, 0.23), *P *=* *0.0001
Physical/sexual IPV	0.68	0.89	0.213, SE* *=* *0.067 (0.08, 0.35), *P *=* *0.0016	0.85	0.75	0.103, SE* *=* *0.099 (−0.09, 0.29), *P *=* *0.30

Notes: WHO, World Health Organization; WAST-Short, Woman Abuse Screening Tool-Short; AUC, area under the curve; AAS, Abuse Assessment Screen; CI, confident intervals; ROC, receiver operating characteristic. Global IPV: having suffered at least one act of emotional, physical or sexual intimate partner violence. Physical/sexual IPV: having suffered at least one act of physical or sexual intimate partner violence.

a AAS emotional is compared with emotional IPV WHO; AAS physical is compared with physical IPV WHO; AAS sexual is compared with sexual IPV WHO; AAS global (emotional, physical or sexual) is compared with Global IPV WHO; AAS physical/sexual is compared with physical/sexual IPV WHO (physical and sexual combined).

#### IPV before pregnancy

The area under ROC curve (AUC) for global IPV (emotional, physical or sexual) was 0.69 for the WAST-Short and 0.69 for the AAS (table 2). In addition, the AUC for physical–sexual IPV was 0.66 and 0.87 for the WAST-Short and for the AAS, respectively. Separating the results by type of IPV (table 2), the AUC for emotional, physical and sexual IPV was found to be 0.69, 0.66 and 0.68 for the WAST-Short and 0.67, 0.88 and 0.82 following AAS. The results also revealed that in all the cases the lowest sum of false-positives and false-negatives for these types of IPV was 0.5 (table 2). Given that the screening scores were integral, therefore, 1 could be the most appropriate cut-off point to identify women suffering these kinds of IPV. Using this cut-off, the WAST-Short accurately categorized 58.9%, 55.2% and 58.3% of the victims of emotional, physical and sexual IPV and 73.5%, 68.5% and 67.9% of non-victims (table 2). In addition, the WAST-Short accurately categorized 59.3% and 57.5% of the victims of global IPV and of physical–sexual IPV and 73.3% and 68% of non-victims. The AAS, using this cut-off, accurately categorized 46.7%, 82.8%, 66.7% of the victims of emotional, physical and sexual IPV, as well as 50.9% of the victims of global IPV and 80% of physical–sexual IPV. In terms of non-victims, the AAS was capable of classifying 87.6% (emotional), 94.6% (physical), 98.3% (sexual), 86.5% (global IPV) and 94.3% (physical–sexual IPV) of non-victims (table 2). Finally, the comparison of the ROC curves of both screening tools to detect global IPV exposure before pregnancy (physical, sexual or emotional) showed no significant difference (*P *=* *0.99) between the WAST-Short and the AAS (table 3). The comparison of the ROC curves for each type of IPV before pregnancy is shown in table 3.

## Discussion

According to the WHO reference standard, in our cohort around a fifth and a tenth of pregnant women suffered at least one act of emotional, physical or sexual IPV before and during pregnancy. Our results indicated that using a cut-off of 1 (instead of 2 as the original version), the WAST-short performed much better than the AAS to detect global and emotional IPV during pregnancy, being an easy and quick-to-answer tool to use in the antenatal care context with just two items. Our results also suggest that it may be necessary to use different screening tools to maximize the detection of IPV before and during pregnancy.

Research conducted in Spain with pregnant women in the postpartum period^7^ reported a slightly higher prevalence of IPV in the 12 months previous to delivery: 4.8%/21% of non-physical abuse and 1.7%/3.6% of physical abuse, according to the AAS and the Index of Spouse Abuse, respectively. We found lower percentages of IPV during pregnancy: 1.8%/9.5% for emotional, 1.2%/1.2% for physical and 0.6%/1% of sexual IPV (according to AAS and WHO questionnaires, respectively).

Related to the screening tools, the WAST has shown good sensitivity, specificity^9^ and psychometric properties.^31^ In Spain, it has shown a sensitivity of 94.5% and specificity of 90.5% in women who attended primary care and domestic violence centres^32^ and its short version (in women who attended primary care) presented a sensitivity of 91.4% and specificity of 76%.^22^ The sensitivity/specificity values found in our sample are similar to those found in other studies with pregnant women: 99.7%/64.4% in Greece,^15^ 66.7–71.5%/89.7% in Japan (short version).^18^

The accuracy of the AAS in pregnancy has exhibited mixed results.^23^ Some studies give adequate figures on its sensitivity (85.7%) to detect physical IPV in pregnant women (midwives’ interviews as reference standard).^19^ It also showed good sensitivity to detect any form of IPV (93%) but low specificity (55%) in a sample of patients (no pregnant women).^33^ One study in Spain using the AAS in pregnant women did not report its screening characteristics.^7^ The Spanish validation in pregnant women^20^ showed very low sensitivity values to detect IPV in the last 12 months: 33.3%, 22.9%, 6.9% (for severe physical abuse, minor psychological abuse and minor physical abuse, respectively), except for severe psychological abuse (sensitivity of 100%). In contrast, in our results the AAS showed a good sensitivity (80%) to detect physical–sexual IPV before pregnancy, but low sensitivity to detect emotional IPV in the same period (46.7%).

The figures of IPV found in this and other studies^1^^,^^7^ show a high enough disease burden to justify screening during pregnancy. WHO does not advise screening in settings where specific resources and appropriate training of healthcare providers are not in place.^34^ However, if resources for women who are exposed to IPV are available, a systematic IPV screening should be highly recommended. The WAST-Short is useful in routinely detecting IPV during pregnancy along with medical, obstetric and family history. The identification of IPV should be useful in establishing a safety plan that is a priority for many women suffering from IPV.^35^ The WAST-short overcomes some barriers such as the shortage of time and difficulty in establishing privacy.^9^^,^^36^ With only two items, which exclude words that refer to violence directly, it reduces discomfort and hesitation concerning IPV screening in both healthcare professionals and women.^37^

Additionally, there is a need in future studies to measure IPV several months after pregnancy to try to disentangle the complex dynamics of IPV in a long-term perspective. There are reports showing greater numbers of violent incidents against women during pregnancy, yet some studies suggest that pregnancy works as a protective event.^16^^,^^26^ Future studies should also consider comparing the ability of screening tools to detect IPV during pregnancy in several time points in the same sample.

### Strengths and limitations

The accuracy analysis presented here deployed two screening tests in a large sample of pregnant women in a prospective study to maximize precision of the findings using paired comparison. Capturing a variety of socioeconomic contexts across 21 centres maximized generalizability. Given the statistically significant difference observed in the comparison of screening tests, our main finding concerning test performance for IPV during pregnancy is reliable. Finally, some studies have investigated the ability of these tools to detect IPV in the 12 months previous to the pregnancy,^20^ but none of them have addressed IPV in the same sample previously and during pregnancy as we did. However, the present research has also some limitation. There was a dropout of participants during the third trimester, which could have reduced the number of pregnant women identified as victims of IPV during pregnancy, affecting precision in the estimation of prevalence.

## Conclusions

Pregnancy represents a uniquely dangerous time for women who become victims of IPV given the potential for both adverse maternal and offspring outcomes. There is an urgent need for detecting and preventing IPV during prenatal visits.^35^ Until recently there was no consensus on routine screening of IPV in all pregnant women,^9^ but this trend is changing as much literature now recommends it.^38–40^ We suggest that the short version of WAST can be used effectively as routine screening to identify IPV during pregnancy and may allow the midwife to initiate a dialog with a possible victimized woman.

## Supplementary data

Supplementary data are available at *EURPUB* online.

## Supplementary Material

ckac009_Supplementary_Data
